# Ti_3_C_2_T_x_MXene Composite 3D Hydrogel Potentiates mTOR Signaling to Promote the Generation of Functional Hair Cells in Cochlea Organoids

**DOI:** 10.1002/advs.202203557

**Published:** 2022-09-18

**Authors:** Zhong Zhang, Shan Gao, Yang‐Nan Hu, Xin Chen, Cheng Cheng, Xiao‐Long Fu, Sha‐Sha Zhang, Xin‐Lin Wang, Yu‐Wei Che, Chen Zhang, Ren‐Jie Chai

**Affiliations:** ^1^ State Key Laboratory of Bioelectronics Department of Otolaryngology Head and Neck Surgery Zhongda Hospital School of Life Sciences and Technology Advanced Institute for Life and Health Jiangsu Province High‐Tech Key Laboratory for Bio‐Medical Research Southeast University Nanjing 210096 P. R. China; ^2^ Department of Biochemistry and Molecular Biology Biomedicine Discovery Institute Monash University Suzhou 215123 P. R. China; ^3^ Shandong Provincial Hospital Shandong First Medical University Jinan 250021 P. R. China; ^4^ Department of Neurobiology School of Basic Medical Sciences Beijing Key Laboratory of Neural Regeneration and Repair Advanced Innovation Center for Human Brain Protection Capital Medical University Beijing 100069 P. R. China

**Keywords:** cochlea organoids, co‐culture, differentiation, functional hair cells, modiolus, MXenes

## Abstract

Organoids have certain cellular composition and physiological features in common with real organs, making them promising models of organ formation, function, and diseases. However, Matrigel, the commonly used animal‐derived matrices in which they are developed, has limitations in mechanical adjustability and providing complex physicochemical signals. Here, the incorporation of Ti_3_C_2_T_x_MXene nanomaterial into Matrigel regulates the properties of Matrigel and exhibits satisfactory biocompatibility. The Ti_3_C_2_T_x_MXene Matrigel composites (MXene‐Matrigel) regulate the development of Cochlear Organoids (Cochlea‐Orgs), particularly in promoting the formation and maturation of organoid hair cells. Additionally, regenerated hair cells in MXene‐Matrigel are functional and exhibit better electrophysiological properties compared to hair cells in Matrigel. MXene‐Matrigel potentiates the amycin (mTOR) signaling pathway to promote hair cell differentiation, and mTOR signaling inhibition restrains hair cell differentiation. Moreover, MXene‐Matrigel facilitates innervation establishment between regenerated hair cells and spiral ganglion neurons (SGNs) growing from the Cochlea modiolus in a co‐culture system, as well as promotes synapse formation efficiency. The approach overcomes some limitations of the Matrigel‐dependent culture system and greatly accelerates the application of nanomaterials in organoid development and research on therapies for hearing loss.

## Graphical abstract

3D Ti_3_C_2_T_x_MXene composited Matrigel (MXene‐Matrigel) has biocompatibility to Cochlear Organoids (Cochlea‐Orgs) and potentiates rapamycin activity to promote differentiation and maturation of Cochlea‐Orgs. Regenerated organoid hair cells are functional and comparable to postnatal day 2 native auditory hair cells. Ti_3_C_2_T_x_MXene also benefits the formation of synapse‐like contacts between regenerated hair cells and sensory neurons.

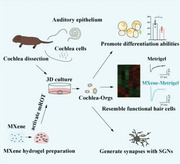

## 1 Introduction

Hearing, balance, and acceleration are senses that are perceived by sensory structures within the inner ear. The epithelium of each of these structures is composed of mechanosensory hair cells and non‐sensory supporting cells. The auditory sensory epithelium in the mouse cochlea is made up of four rows of hair cells and adjacent supporting cells.^[^ ^1^ ^]^ On the apical surface, hair cells have F‐actin‐rich hair bundles composed of organized stereocilia. Mechanotransduction channels located in stereocilia detect mechanical motions induced by physical motion or sound waves and produce graded responses in neurotransmitter release.^[^ ^2^ ^]^ Those signals are sent to the central nervous system (CNS) via associated neurons innervated by branches of the VIIIth nerve. In nonmammalian vertebrates such as zebrafish and birds, the loss of auditory hair cells causes surrounding supporting cells to either immediately transdifferentiate into hair cells, or dedifferentiate into hair cells and supporting cells following rounds of cell division.^[^ ^3^ ^]^ Unlike birds, once human inner ear hair cells are lost, they cannot regenerate in vivo.^[^ ^4^ ^]^ Sensorineural hearing loss is currently one of the most frequent health problems, especially in the aged, and it is mainly caused by the degradation of cochlea hair cells.^[^ ^5^, ^6^ ^]^

Stem cells have been discovered in the cochlea sensory epithelium of the postnatal mice,^[^ ^7^, ^8^, ^9^ ^]^ which have the ability to proliferate and differentiate into particular cell types, including hair cells and supporting cells. And damage or Wnt signaling activation can potentiate the proliferation abilities of cochlea stem cells.^[^ ^9^, ^10^ ^]^ Recently, in the 3D culture system, stem cells from various tissues and organs, including the cochlea, have been able to multiply and differentiate into mini‐organs with a variety of cell types, which were also known as organoids.^[^ ^11^, ^12^ ^]^ Using induced pluripotent stem cells, embryonic stem cells, and a small group of tissue‐specific resident stem/progenitor cells, Cochlea‐Orgs have been successfully developed into 3D structures in vitro.^[^ ^13^, ^14^, ^15^ ^]^ Organoids show some characteristics of real organs, including cellular composition and physiological characteristics, making them ideal models for the research of organ development, function, and diseases.^[^ ^13^ ^]^ However, the commonly used matrices for organoids culture, such as Matrigel and Collagen, have difficulties in biomimicking extracellular environments of different tissues and providing complicated physicochemical signals.^[^ ^16^ ^]^

Nanomaterials are frequently put into scaffolds to enhance their characteristics, for example, graphene‐enhanced electrical conductivity of incorporated hydrogels.^[^ ^17^ ^]^ MXenes is a class of metal carbide or metal nitride nanomaterial having a 2D layered structure.^[^ ^18^ ^]^ Mn+1X+nTx (n = 1–3) is the formula for all of these MXenes, where M is an early transition metal (Sc, Ti, V, Cr, Zr, Nb, Mo, Hf, etc.), X is carbon and/or nitrogen, and Tx is a surface functional group (‐O, ‐OH, etc.).^[^ ^19^, ^20^ ^]^ MXenes have excellent electrical conductivity because of their metallic backbone and inherit the hexagonal lattice symmetry of their parent MAX phase.^[^ ^21^ ^]^ Many functional groups on the surface of MXenes give a large number of active sites, which provides significant potential for surface modification and exceptionally effective loading of active molecules.^[^ ^22^ ^]^ MXenes also have good thermal conductivity, an adjustable bandgap, and high mechanical strength, making them promising candidates for energy storage, sensing, environmental protection, and other applications.^[^ ^20^, ^23^ ^]^ In our previous research, we found that the Ti_3_C_2_T_x_MXene film enhanced neuronal development, resulting in neuron differentiation and maturation, especially the formation and maturation of synapses.^[^ ^24^ ^]^

Mammalian target of rapamycin (mTOR) detects nutrition and growth factors to modulate metabolism, autophagy, cell proliferation, and stem cell differentiation.^[^ ^25^ ^]^ Recently, Ti_3_C_2_T_x_MXene has been reported to activate WNT/HIF‐1*α*‐Mediated metabolism and induce osteogenic differentiation of human periodontal ligament cells.^[^ ^26^ ^]^ Hypoxia‐inducible factors‐1*α* (HIF‐1*α*) transcription is enhanced by mTOR activity, while the addition of mTOR inhibitor rapamycin can eliminate many of the effects mediated by the expression of HIF‐1*α*.^[^ ^27^ ^]^ mTOR activity has been reported to participate in the maintenance and differentiation of stem cells, including neural stem cells,^[^ ^28^ ^]^ hematopoietic stem cells,^[^ ^29^ ^]^ and germ stem cells.^[^ ^30^ ^]^ mTOR signaling has been also found to regulate cochlear progenitor cells to de‐differentiate and generate hair cells.^[^ ^14^ ^]^ We previously found that the formation and long‐term survival of hair cells were affected in mTOR‐deficient mice.^[^ ^31^ ^]^ Therefore, mTOR/ HIF‐1*α* signaling may participate in MXene‐induced organoid hair cell differentiation.

Herein, we constructed a 3D hydrogel culture system by incorporating an appropriate proportion of Ti_3_C_2_T_x_MXene into Matrigel and investigated its effects on the development and maturation of Cochlea‐Orgs. We found that MXene‐Matrigel maintained the self‐renewal abilities of Cochlea‐Orgs and significantly promoted hair cell differentiation and maturation. We proved that the regenerated hair cells are functional, and comparable to native hair cells by recoding their electrophysiological properties. We demonstrated that MXene‐Matrigel promoted hair cell differentiation by activating mTOR signaling. We also described a co‐culture system of Cochlea‐Orgs and modiolus and successfully established innervations of regenerated hair cells. Furthermore, in our co‐culture system, MXene‐Matrigel improved the efficiency of synapse formation relative to Matrigel, making it promising for the application to in vivo synaptic reconstruction.

## 2 Results

### 2.1 Preparation and Characterization of Ti_3_C_2_T_x_ Mxene

**Figure 1:**
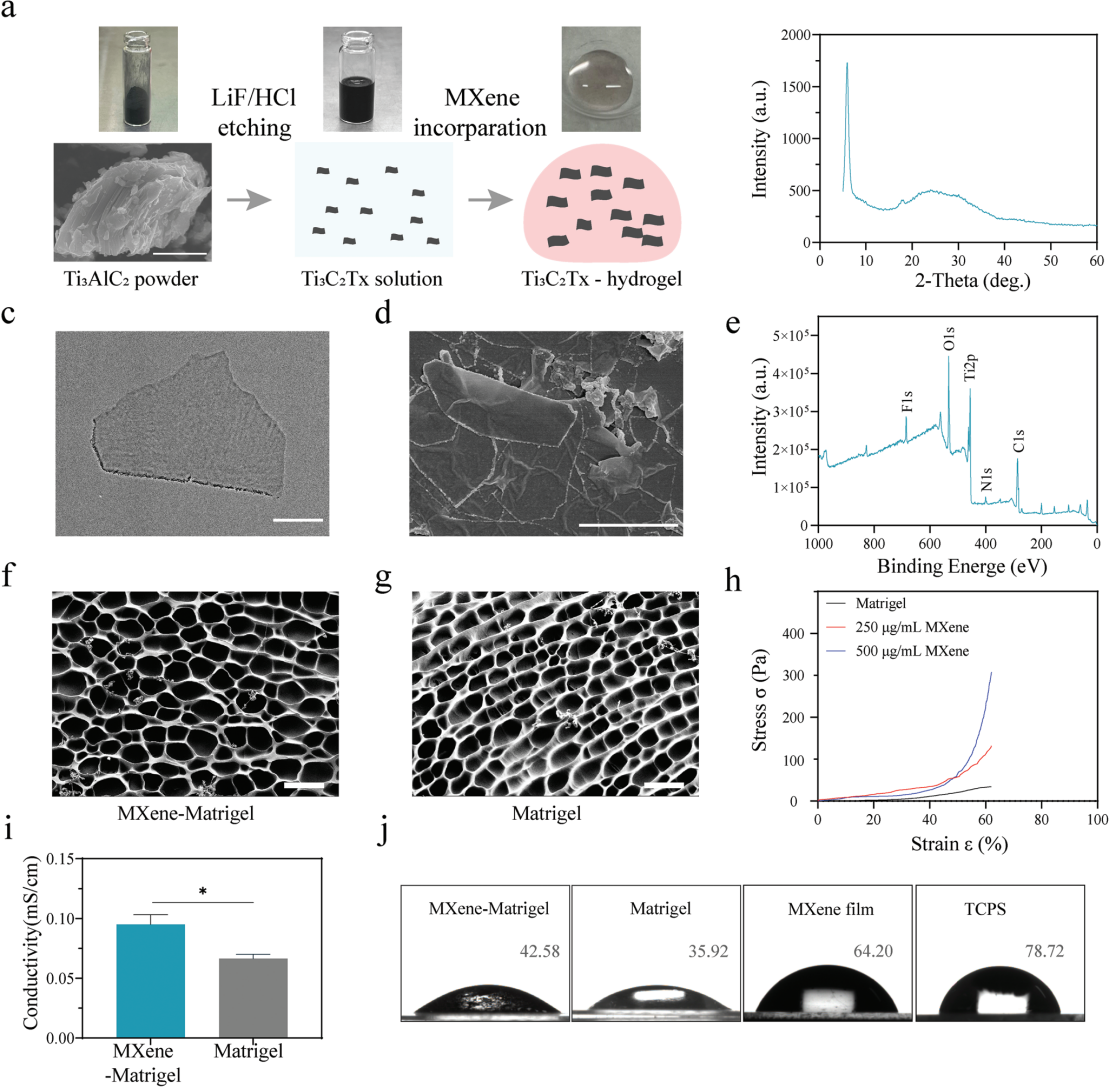
Ti_3_C_2_TxMXene and MXene‐Matrigel hydrogel preparation and characterization. a) Schematic diagram of the preparation of Ti_3_C_2_TxMXene and MXene‐Matrigel. Appropriate amount of Ti3C2TxMXene solution was mixed with Matrigel, and the incorporating hydrogel was solidified at 37 °C. b) The XRD pattern of Ti_3_C_2_T_x_MXene. The characteristic peak of Ti_3_C_2_T_x_MXene was prior to 8°. c) Representative TEM image of the Ti_3_C_2_T_x_MXene nanosheets. Scale bar = 200 nm. d) SEM image of the Ti_3_C_2_T_x_MXene solution, which was diluted to 100 µg mL^−1^ by water. Scale bar = 2 µm. e) XPS patterns of the Ti_3_C_2_T_x_MXene film. Abundant functional groups containing O, Ti, and C were detected. f,g) SEM images of MXene‐Matrigel (f) and Matrigel (g). Scale bar = 50 µm. h) Stress‐strain curve of Matrigel and MXene‐Matrigel (250 µg mL^−1^ Ti_3_C_2_T_x_MXene, red; 500 µg mL^−1^ Ti_3_C_2_T_x_MXene, blue). i) The conductivity of Matrigel and MXene‐Matrigel (250 µg mL^−1^). (Data are presented as mean ± SD, n = 3, * *p* < 0.05). j) The water contact angles of MXene‐Matrigel (250 µg mL^−1^), Matrigel, Ti_3_C_2_TxMXene film, and TCPS.

The preparation of the Ti_3_C_2_T_x_MXene is shown in the schematic diagram (**Figure** **1**a). The Ti_3_C_2_T_x_MXene solution was prepared as previously described.^[^ ^24^ ^]^ Briefly speaking, Ti_3_C_2_T_x_MXene was produced by Al‐selective etching of Ti_3_AlC_2_ (MAX) phase in a mixture of HCl and LiF. A typical X‐ray diffraction (XRD) pattern of the Ti_3_C_2_T_x_MXene film is shown in Figure 1b. Prior to 8°, the characteristic peak matched the (002) plane of Ti_3_C_2_TxMXene.^[^ ^20^ ^]^After the Ti_3_C_2_T_x_MXene solution was diluted with deionized water, ultrasound was performed and dropped onto a carbon‐coated‐copper grid for transmission electron microscopic imaging (TEM). The TEM images exhibited the typical structure of the Ti_3_C_2_T_x_MXene sheet and showed a single layer Ti_3_C_2_T_x_MXene with a diameter of hundreds of nanometers (Figure 1c). To examine the surface morphology characteristics of Ti_3_C_2_T_x_MXene in a 3D environment, the scanning electron microscope (SEM) was used, and the imaging results showed that Ti_3_C_2_T_x_MXene presented a single or multi‐layer structure in a 3D solution (Figure 1d). Next, to analyze the surface chemistry of Ti_3_C_2_T_x_MXene, X‐ray electron energy spectroscopy (XPS) was used to detect the surface characteristics. The results (Figure 1e) showed that Ti_3_C_2_T_x_MXene is primarily composed of C, O, Ti, and F, with abundant functional groups on the surface.

### 2.2 Preparation and Characterization of Mxene‐Matrigel

**Figure 2:**
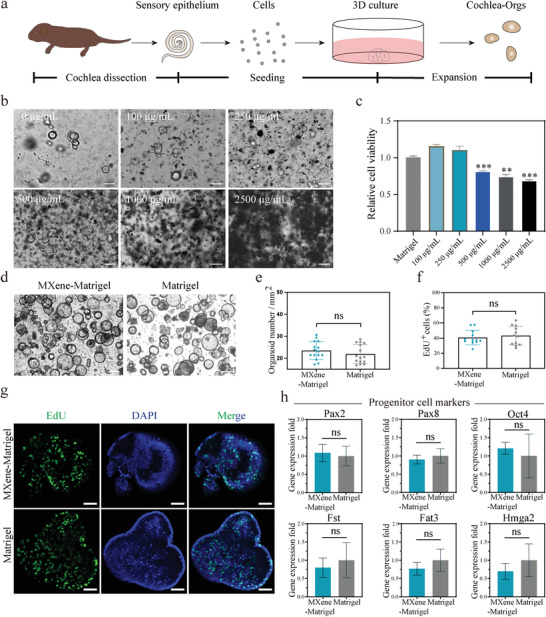
MXene‐Matrigel maintain proliferation abilities of Cochlea‐Orgs. a) Schematic illustration of the isolation and culture of primary cochlea cells and the formation of Cochlea‐Orgs. b) After proliferation for 7 days, Ti3C2TxMXene concentration screening was conducted in a dose‐response manner. 0–2500 µg mL^−1^ MXene was incorporated into Matrigel to evaluate toxicity for cochlea cells. Scale bar = 100 µm. c) The relative cell viability of Cochlea‐Orgs cultured in different concentrations of MXene‐Matrigel for 7 days was detected by CCK‐8 kit. (Data are shown as mean ± SD, n = 3, from one representative experiment, and three independent experiments) * * *p* < 0.01, *** *p* < 0.001. d) BF images of Cochlea‐Orgs at day 10 cultured in Matrigel and MXene‐Matrigel. Scale bar = 100 µm. e) Number of organoids formed per square millimeter from an equal number of cochlea cells. (Data are shown as mean ± SD, n = 15, three independent experiments). f) Percentages of EdU+ cells in Cochlea‐Orgs of the Matrigel group and the MXene‐Matrigel group. (Data are shown as mean ± SD, n = 12, three independent experiments). g) Confocal images of EdU labeling proliferation of Cochlea‐Orgs. EdU (green), DAPI (blue). Scale bar = 50 µm. h) qRT‐PCR analysis of gene expression of cochlea progenitor cells markers in the MXene‐Matrigel group relative to the Matrigel group (Experiments were performed in independent experiments. Data are shown as mean ± SEM, n = 3).

To verify whether Ti_3_C_2_T_x_MXene could regulate the properties of Matrigel, we prepared 3D hydrogel composites by incorporating Ti_3_C_2_T_x_MXene into Matrigel (Figure 1a and **Figure** **2**b). First, the concentration of MXene was adjusted to 250 µg mL^−1^ through screening (Figure 2b). SEM images of MXene‐Matrigel and Matrigel were similar (Figure 1f,g), confirming that the MXene nanomaterial cannot be identified on the micro‐scale of cell size. Figure 1h illustrates that MXene incorporation modulated the strength of Matrigel. As the concentration increased from 0 to 500 µg mL^−1^, the compressive stress at a strain of 60% increased from 30 to 330 Pa, which was comparable to the compressive stress of Matrigel.^[^ ^32^ ^]^ As shown in Figure 1i, the conductivity of Matrigel was between a range of 0.0608 to 0.0733 mS cm^−1^, while the conductivity of the MXene‐Matrigel composites was significantly higher than that of Matrigel. It proved that the incorporation of Ti_3_C_2_T_x_MXene enhanced the conductivity of Matrigel. The hydrophilicity of MXene‐Matrigel, Matrigel, and Ti_3_C_2_TxMXene film was detected by water contact angle measurements (Figure 1j). The contact angles of the Ti_3_C_2_TxMxene film and tissue culture polystyrene (TCPS). Due to the high hydrophilicity of MXene, the hydrophilicity of Matrigel was increased by MXene incorporation (300 µg mL^−1^).

### 2.3 Biocapacity of MXene‐Matrigel

As shown in the schematic diagram (Figure 2a), cochlea cells were isolated from the cochlea of around postnatal day 2 (P2) wild‐type C57BL/6 mice by collagenase digestion, followed by cell suspension and seeding to Matrigel or the MXene‐Matrigel composites in an equal number. In the cochlea of postnatal mice, there are some Lgr5+ progenitor cells.^[^ ^33^ ^]^ And A variety of growth factors and small molecules were used to support the proliferation of Lgr5+ progenitor cells and the generation of Cochlea‐Orgs, including EGF, FGF, CHIR99021, VPA, PVc, and A83‐01.^[^ ^8^, ^12^ ^]^ Although Ti_3_C_2_T_x_MXene has been shown biocompatible for neuron culture in a 2D culture system,^[^ ^24^ ^]^ the toxicity for cochlea cells remains unclear.

Cochlear hair cells are sensitive to various factors, such as manganese ion, neomycin, and cisplatin, which can damage cochlea hair cells both in vivo and in vitro.^[^ ^5^ ^]^ To evaluate the toxicity of MXene, a wide range of MXene concentrations was tested in a dose‐dependent manner (0 to 2500 µg mL^−1^) (Figure 2b). After 7 days of proliferation, Cochlea‐Orgs emerged from stem cells cultured in full complete medium (FCM). And Cell Counting Kit‐8 (CCK8) assay was conducted to calculate the total cell number in hydrogel (Figure 2c). It showed that 0–250 µg mL^−1^ Mxene did not influence the relative cell viability of Cochlea‐Orgs. However, when the concentration of MXene increased to 500 µg mL^−1^, the development of Cochlea‐Orgs was significantly affected, and smaller pellets were formed in the MXene‐Matrigel hydrogel. And when the concentration of MXene increased to 2500 µg mL^−1^, it became difficult for hydrogel to solidify and only a few cells could survive. According to this screening and further test (data not shown), an optimized concentration of Mxene (300 µg mL^−1^) was used for the subsequent experiments.

Next, the toxicity of MXene has also been measured at different time points (Figure S1a, Supporting Information). After incubation with CCK8, the absorbance at 450 nm (A450) was measured to detect the relative cell number of organoids in the Mxene‐Matrigel (300 µg mL^−1^) and the Matrigel groups on day 10 (Figure S1b,c, Supporting Information). Normalized A450 of the MXene‐Matrigel group was about 1.08‐fold in comparison to the Matrigel group, confirming that the relative cell viability of the MXene‐Matrigel group had no significant difference from the Matrigel group. The cell number of the MXene‐Matrigel group was equal to the Matrigel group, which showed that MXene‐Matrigel maintained the proliferation abilities of Cochlea‐Orgs.

### 2.4 MXene‐Matrigel Maintained Proliferation Abilities of Cochlea‐Orgs

The relative cell viability indicated similar proliferation abilities of Cochlea‐Orgs in MXene‐Matrigel and Matrigel (Figure 2b,c). After 10 days of expansion, Cochlea‐Orgs maintained a similar appearance in the MXene group and the MXene‐Matrigel group (Figure 2d). Organoids forming efficiency was about 16.80% to 28.56% in the Matrigel group and comparable to that of the MXene‐Matrigel group, which was about 17.22% to 30.66% (Figure 2d,e). To label proliferating cells, confocal images of the Edu labeling (green) to identify the proliferation abilities of Cochlea‐Orgs (Figure 2e–g). After 10 days of expansion, organoids of the MXene‐Matrigel group exhibited an equivalent proportion of EdU+ cells (Figure 2f) compared to organoids cultured in Matrigel. In the Matrigel group, the percentage of Edu+ cells was 42.35 ± 2.44. And in the MXene‐Matrigel group, the percentage of Edu+ cells was 43.20 ± 5.04, which has no significant difference from the Matrigel group.

To address whether MXene‐Matrigel maintained the self‐renewal abilities of progenitor cells, qPCR was performed to analyze the expression of early optic markers, Pax2 and Pax8,^[^ ^34^ ^]^ the prosensory cell‐specific markers, Fst, Fat3 andHmga2,^[^ ^35^ ^]^ and stem cell marker, Oct4^[^ ^36^ ^]^ (Figure 2f). The expression of these genes was not significantly changed in organoids cultured in MXene‐Matrigel compared to control, whereas the mRNA expression levels of Fst, Fat3, Hmga2, and Pax8 were slightly higher in the Matrigel group and the mRNA levels of Oct4, and Pax2 in the Matrigel group were slightly lower in the MXene‐Matrigel group.

### 2.5 MXene‐Matrigel Promoted Hair Cells Formation of Cochlea‐Orgs

**Figure 3:**
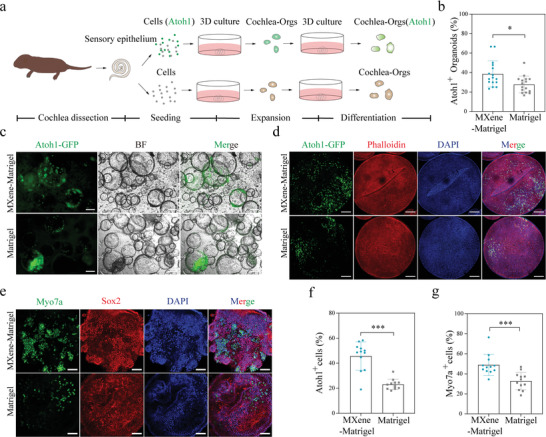
MXene‐Matrigel potentiated hair cells formation of Cochlea‐Orgs. a) Overview of the generation of cochlea hair cells through the differentiation of Cochlea‐Orgs. b) Atoh1‐GFP+ organoids forming efficiency of the MXene‐Matrigel group and the Matrigel group (n = 16, data are from four independent experiments). c) BF and green fluorescent (Atoh1‐GFP) images of Cochlea‐Orgs after 20 days of differentiation in the differentiation medium. Scare bar = 100 µm. d) Confocal images of DAPI (blue), early hair cell marker Atoh1‐GFP, and phalloidin (red). Scare bar = 100 µm. e) Confocal images of DAPI (blue), hair cell marker Myo7a (green), and supporting cell marker Sox2 (red). Scare bar = 50 µm. f) Percentages of Atoh1‐GFP+ newly generated hair cells of Cochlea‐Orgs after 10 days of proliferation in the full completed medium (n = 12, data are from three independent experiments). g) Percentages of Myo7a+ hair cells of Cochlea‐Orgs after additional 20 days of differentiation. Experiments were performed in independent experiments. Data are shown as mean ± SD, * Indicates *p* < 0.05. *** indicates *p* < 0.001.

Atoh1 is an early key regulator for hair cell generation and is applied as a marker for newly generated hair cells.^[^ ^37^ ^]^ After 10 days of expansion, Atoh1‐GFP+ hair cells emerged in Cochlea Orgs. Whereas another hair cell marker, Myo7a, failed to detect most of the Atoh1‐GFP+ hair cells (Figure S2, Supporting Information), which proved that these Atoh1‐GFP+ hair cells were not preexisting but regenerated. To verify and trace newly generated hair cells in organoids, the Atoh1‐GFP mouse line and Wild‐type mice were used for the culture of Cochlea‐Orgs (**Figure** **3**a). After 10 days of expansion, the percentage of Atoh1‐GFP+ organoids was higher in the MXene‐Matrigel group, indicating differentiation abilities of Cochlea‐Orgs were enhanced by incorporating of MXene (Figure 2b). Furthermore, fluorescent images exhibited significantly higher hair cell production efficiency in the MXene‐Matrigel group compared to the Matrilgel group (Figure 3c), illustrating that MXene‐Matrigel promoted the formation of hair cells. Furthermore, confocal images were taken to examine the efficiency of hair cell production (Figure 3d). The percentage of Atoh1‐GFP+ hair cells in the MXene‐Matrigel group was significantly higher than that in the Matrigel group. The statistical results demonstrated that Atoh1‐GFP+ organoid hair cells accounted for about 22.90% of the total DAPI labeled cells in the Matrigel group, while the proportion of Atoh1‐GFP+ newly generated organoid hair cells in the MXene‐Matrigel group was about 48.33% (Figure 3f).

**Figure 4:**
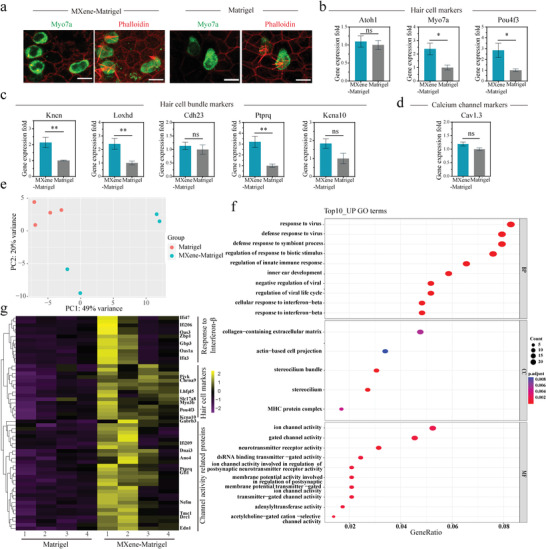
Characterization of mouse Cochlea‐Orgs cultured in Matrigel and MXene‐Matrigel. a) Representative images of organoid hair cells in the Mxene‐Matrigel group and the Matrigel group. Single plane (left) and Z‐stack (right) confocal images of organoid hair cells cultured in Mexne‐Matrigel and Matrigel. Hair cells were labeled by Myo7a (green), and actin‐rich hair cell bundles were labeled by Phalloidin (red). Scale bar = 10 µm. b–d) q‐PCR analysis of relative gene expression of hair cell specific markers: Hair cell markers (b), hair cell bundle markers (c), and calcium channel marker (d) of Cochlea‐Orgs cultured in the MXene‐Matrigel relative to organoids in the Matrigel (Data are shown as mean ± SEM. n≥6, * Indicates *p* < 0.05. ** indicates *p* < 0.01, *** indicates *p* < 0.001). e,f) mRNA‐seq analysis of Cochlea‐Orgs in MXene‐Matrigel and Matrigel (n = 4, from four independent wild‐type mice). (e) PCA plot showing the clustering of Cochlea‐Orgs in the two groups. (f) GO enrichment of Cochlea‐Orgs in the MXene‐Matrigel group compared to the Matrigel group. g) Heatmap of Cochlea‐Orgs gene expression obtained by mRNA sequencing comparing four independent Cochlea‐Orgs grown in MXene‐Matrigel with organoids grown in Matrigel.

Next, we extended the differentiation time of organoid to additional 20 days in the differentiation medium. Since the expression level of Atoh1 gradually decreased during the maturation of hair cells in the cochlea, it is difficult to analyze the number of mature hair cells in organoids by Atoh1. Thus, Myo7a, the most used marker of hair cells,^[^ ^13^ ^]^ was applied to label hair cells in subsequent experiments (Figure 3e,g, **Figure** **4**a and Figure 6a–d). Figure 3e gives confocal images stained for the hair cells marker Myo7a (green) and supporting cells marker (sox2) and Phalloidin (red), confirming that Cochlea‐Orgs were composed of hair cells and supporting cells, which were the same as the sensory basilar membrane of the cochlea. Moreover, the proportion of Myo7a+ cells in the two groups was counted (Figure 3g). The results showed that the proportion of Myo7a+ hair cells in organoids cultured in the MXene‐Matrigel was significantly higher than that in the Matrigel substrate: 49.90% of the MXene‐Matrigel substrates and 30.73% of the Matrigel substrates. These data demonstrated that the MXene‐Matrigel composites promoted the regeneration of hair cells in the Cochlea‐Orgs.

### 2.6 Characterization of Cochlea‐Orgs

The morphology of organoid hair cells (Myo7a, green) in MXene‐Matrigel and Matrigel were similar, whereas hair bundles of hair cells were more organized in MXene‐Matrigel. (Figure 4a). To further characterize Cochlea‐Orgs in the two groups, qPCR analysis was conducted in independent experiments in the same condition to analyze the expression of the specific markers of hair cells, including the hair cell markers Myo7a, Atoh1, and Pou4f3 (Figure 4b), hair bundle markers Kncn, Loxhd1, Cdh23, Ptprq, and kcna10 (Figure 4c), and calcium channel marker Cav1.3 (Figure 4d). The significantly higher expression level of hair bundle marker, Kncn, Loxhd1, Ptprq, and hair cell marker, Myo7a, Pou4f3 demonstrated that MXene‐Matrigel facilitated the maturity of hair bundles of organoid hair cells. It was worth pointing out that there was no statistical difference in the mRNA expression level of the early hair cell marker Atoh1, which was consistent with the results that the expression of Atoh1‐GFP gradually decreased (Figure S2, Supporting Information).

Next, mRNA sequencing was performed to analyze the changes in mRNA expression induced by MXene incorporation from three independent experiments (Figure 4e–g). The principal component analysis (PCA) plot underscored the difference between Cochlea‐Orgs cultured in MXene‐Matrigel and Matrigel (Figure 4e). Moreover, the differentially expressed genes in Cochlea‐Orgs grown in MXene‐Matrigel and Matrigel were analyzed using GO enrichment analysis. Furthermore, GO enrichment analysis of different expression genes was performed at the levels of biological processes (BP), cellular components (CC), and molecular functions (MF). Native hair cells are functional in detecting movement induced by sound waves through cilium of hair bundles, followed by neurotransmitter release to innervated SGNs.^[^ ^38^ ^]^ Figure 4f exhibits the top up‐regulated GO terms of the two groups, and GO terms are up‐regulated including inner ear development, response to interferon‐beta, stereociliun, channel activity, and neurotransmitter receptor activity, which are crucial for the differentiation and function of hair cells. The GO results matched with the above histomorphology results (Figure 3e,d and Figure 4a), confirming that MXene‐Matrigel promoted organoid hair cell differentiation and predicting newly formed hair cells seem to more mature than organoid hair cells in Matrigel. Figure 4g represents a heatmap of the expression of hair cell marker genes (Pou4f3, Slc17a8, known asVglut3, Myo3b, and kcna10),^[^ ^14^ ^]^ channel‐related genes (TMC1, Ano4, Ptprq, and Gfi1)^[^ ^39^ ^]^ and interferon related genes (Ifi206, Zbp1, and Oas1a). Notably, the differentiation‐related genes were up‐regulated in the MXene‐Matrigel group, revealing MXene‐Matrigel promoted hair cell differentiation. These results were consistent with q‐PCR data (Figure 6b–d).

### 2.7 Electrophysiological Properties of Regenerated Hair Cells in Cochlea‐Orgs

**Figure 5:**
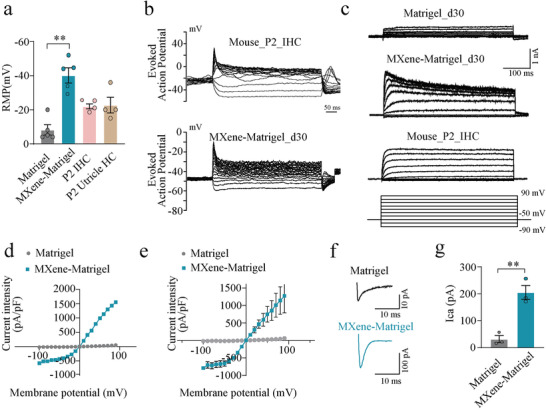
Functional organoid hair cells in MXene‐Matrigel exhibited better electrophysiological properties. a) Statistic results of RMP of regenerated organoid hair cells in the Matrigel and the MXene‐Matrigel substrates after differentiation for 20 days (d30), P2 native IHCs, and P2 utricle hair cells. (n ≥4, from one representative experiment, three independent experiments, **p < 0.01. b) Representative evoked spikes recorded under current‐clamp, from native (WT) IHCs at P2 and organoid hair cells (d30). Evoked spikes and voltage responses were recorded in response to step current injection (from −30 to 160 pA, ms,10 pA per step). c) Representative K+ currents recorded by the protocol shown below. d,e) The I–V plot of averaged the fast component (d) and low component (e) of the K+ currents corresponding to (c) (n≥3, from one representative experiment, two independent experiments). f) Representative calcium currents (*I*_ca_) recorded in response to a voltage ramp from −87 to +63 mV in 150 ms under voltage clamp, from organoids hair cells of the Matrigel group or the MXene‐Matrigel group at d30. g) Statistic results of the peaks of *I*_ca_, recorded from organoid hair cells of the Matrigel group or the MXene‐Matrigel group at d30. (n = 3, from one representative experiment, two independent experiments) * Indicates *p* < 0.05. ** indicates *p* < 0.01, *** indicates *p* < 0.001.)

To investigate the maturity of regenerated cochlear hair cells, electrophysiological recordings were conducted using a whole‐cell patch‐clamp to compare the function and similarity of these newly generated hair cells to native hair cells. Here, using single‐cell electrophysiology, the functional properties of newly generated organoid hair cells, which were derived from the Atoh1‐GFP mouse line, were assessed after 20 days of differentiation at d30 (**Figure** **5**). The resting membrane potential (RMP) for organoid hair cells cultured in MXene‐Matrigel was −40.17 ± 4.446 mV (mean ± SD), which was significantly lower than the Matrigel group (−8.200 ± 3.040 mV), also lower than P2 cochlear inner hair cells (IHCs) (−22.00 ± 1.422 mV) and P2 vestibular hair cells (−22.75 ± 4.571 mV) (Figure 5a). Voltage responses (Figure 5b) and current responses (Figure 5c) of organoid hair cells were also recorded. And it was found that organoid hair cells acquired the capability to generate action potentials (APs) in response to membrane depolarization. Furthermore, the voltage responses of organoid hair cells were similar to P2 native IHCs (Figure 5b) and to P2 utricle hair cells.^[^ ^40^ ^]^ Organoid hair cells in organoids (d30) in the MXene‐Matrigel group exhibited similar outward rectifying K+ currents as P2 native IHCs (Figure 5c), which is significantly higher than the Matrigel group. Organoid hair cells showed typical I–V curves of averaged the fast component (Figure 5d) and low component (Figure 5e) of potassium currents, both of them were better than the Matrigel group. Moreover, calcium currents were also observed in the d30 organoid hair cells (Figure 5f). The statistical results showed that the calcium currents of the MXene‐Matrigel group (205.4 ± 25.58 pA) were significantly bigger than the Matrigel group (31.57 ± 13.01 pA) (Figure 5g). Taken together, these data revealed that several characteristics of hair cells in the MXene‐Matrigel group were better than the Matrigel group, illustrating that electrophysiological properties of regenerated hair cells in MXene‐Matrigel are at least comparable to P2 native IHCs.

### 2.8 mTOR‐HIF1*α* Signaling Pathway was Activated in the MXene‐Matrigel Hydrogel

**Figure 6:**
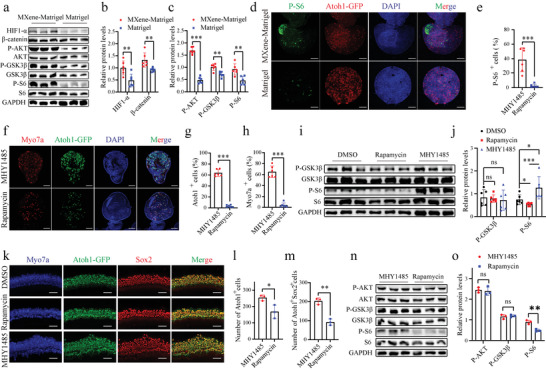
MXene‐Matrigel potentiated mTOR signaling pathway to promote hair cell differentiation. a‐d) MXene‐Matrigel enhanced mTOR signaling pathway of Cochlea‐Orgs. a) Immunoblots for HIF‐1*α*, *β*‐catenin, P‐AKT, AKT, P‐GSK3*β*, GSK3*β*, P‐S6, S6, and GAPDH using protein lysates of organoids cultured in MXene‐Matrigel and Matrigel after 5 days of differentiation. (n = 3, from one representative experiment, two independent experiments). b) Relative protein levels of HIF‐1*α* and *β*‐catenin were compared to GAPDH (n = 6, two independent experiments). c) Relative protein levels of P‐AKT, P‐GSK3*β*, and P‐S6 were quantified by comparing to AKT, GSK3*β*, and S6, respectively (n = 6, two independent experiments). d) Confocal images showed increased P‐S6 expression (red) in organoids of the MXene‐Matrigel group. Scale bar = 50 µm. e) Proportion of P‐S6+ cells in organoids (n = 6, two independent experiments). f–j) Hair cell formation was affected by regulating mTOR singling of organoids. (f) Confocal images of organoids cultured in the presence of mTOR activator MHY1485, mTOR inhibitor rapamycin, and DMSO control. Scale bar = 50 µm. Newly generated hair cells are labeled by Myo7a (red) and Atoh1‐GFP (green). Proportion of Myo7a+ cells (g) and Atoh1‐GFP+ hair cells (h) in organoids after 5 days of treatment of MHY1485 and rapamycin (n = 6, two independent experiments). (i) Immunoblots for P‐AKT, AKT, P‐GSK3*β*, GSK3*β*, P‐S6, S6, and GAPDH using protein lysates of organoids cultured in MXene‐Matrigel and Matrigel after 5 days of differentiation (n = 3, from one representative experiment, three independent experiments). (j) Relative protein levels of P‐GSK3*β* and P‐S6 were quantified, respectively (n = 6, two independent experiments). k–m) Hair cell formation was affected by regulating mTOR singling of cochlear explants (n = 3, from independent mouse cochlear explants). (k) Confocal images of middle turn of MHY1485, rapamycin, and DMSO‐treated cochlear explants. Number of Atoh1+ hair cells (l) and Atoh1+ Sox2+ newly formed hair cells (m) after 4 days of treatment of MHY1485 and rapamycin. Scale bar = 50 µm. n) Immunoblots for P‐AKT, AKT, P‐GSK3*β*, GSK3*β*, P‐S6, S6, and GAPDH using protein lysates of cochlear explants after 4 days of treatment of MHY1485 and rapamycin (n = 3, from independent mouse cochlear explants). o) Relative protein levels of P‐AKT, P‐GSK3*β*, and P‐S6 were quantified, respectively (n = 3, from independent mouse cochlear explants).

It has been previously reported that MXene promoted the differentiation of human periodontal ligament cells through activating WNT/HIF1‐*α* signaling.^[^ ^26^ ^]^ Recently, mTOR signaling has been discovered to participate in the differentiation and long‐term survival of hair cells.^[^ ^14^, ^31^ ^]^ Here, we found some of the top 10 up‐regulated GO terms were related to mTOR signaling, particularly the cell response to interferon‐beta (Figure 4f). Commonly used indicators for mTOR activity are P‐AKT, P‐GSK3*β*, and P‐S6.^[^ ^31^ ^]^ And *β*‐catenin is an indicator of WNT activity.^[^ ^41^ ^]^ The protein expression levels of HIF‐1*α*, *β*‐catenin, P‐AKT, AKT, P‐GSK3*β*, GSK3*β*, P‐S6, and S6 were examined in organoids cultured in MXene‐Matrigel and Matrigel (**Figure** **6**a–c). It showed that mTOR activity and WNT activity was up‐regulated in organoids cultured in MXene‐Matrigel. Furthermore, the percentage of P‐S6+ cells in organoids of the MXene‐Matrigel group is significantly higher (Figure 6d,e).

To determine whether mTOR activity are crucial for hair cell formation in organoids, mTOR activity was potentiated or attenuated by agonist MHY1485 and antagonist rapamycin, respectively (Figure 6f–j). The percentage of Myo7a+ and Atoh1‐GFP+ cells in organoids was calculated after treatment of MHY1485, rapamycin, or DMSO for 5 days (Figure 6g,h). After mTOR agonist or antagonist treatment, hair cell formation was significantly affected, confirming the essential roles of mTOR activity in hair cell differentiation. Moreover, the protein expression levels of P‐GSK3*β*, GSK3*β*, P‐S6, and S6 were examined in organoid (Figure 6i,j), which verified mTOR signaling was regulated by MHY1485 and rapamycin.

Next, we analyzed whether mTOR activity is crucial for hair cell formation in the auditory epithelium. Cochlear explants were treated with MHY1485 or rapamycin for 4days before examining the percentage of Atoh1‐GFP+ cells or Atho1+ Sox2+ newly formed hair cells (Figure 6k–m). Similar to Cochlea‐Orgs, the proportion of hair cells is up‐regulated or down‐regulated after treatment with MHY1485 or rapamycin, respectively. To verify the activity of mTOR, the protein level of P‐AKT, P‐GSK3*β* and P‐S6 was examined (Figure 6n,o).

### 2.9 MXene‐Matrigel Promoted the Formation of Synapse‐like Contacts between Hair Cells and Sensory Neurons

**Figure 7:**
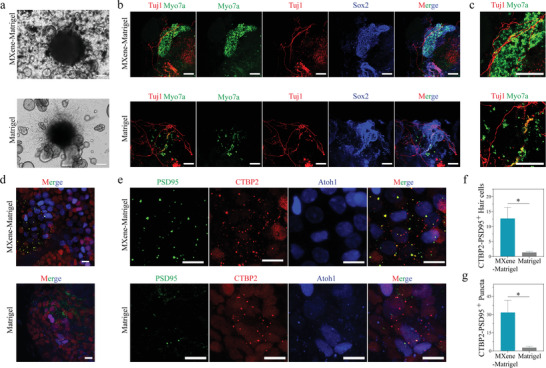
Cochlea‐Orgs co‐cultured with modiolus and form synapse‐like contacts with sensory neurons. a) BF images showed the co‐culture of Cochlea‐Orgs and modiolus in MXene‐Matrigel and Matrigel. b,c) Representative 3D projection images showed Myo7a+ (green) hair cells and Sox2+ supporting cells were surrounded by clusters of Tuj1+ sensory‐like neurons in a whole mount immunostained sample cultured in MXene‐Matrigel and Matrigel. Representative region of interest was highlighted (c). Neuron marker Tuj1 (red), hair cell marker Myo7a (green), supporting cell marker Sox2 (blue). Scare bar = 100 µm. d,e) Representative confocal images presented co‐localization of CTBP2+ and PSD95+ puncta. A dotted region of interest in the inset shows the putative synapses (e). PSD95 (green), CTBP2 (red), hair cell marker Atoh1 (blue). Scare bar = 10 µm. f) The number of CTBP2+PSD95+ hair cells in the two groups. g) The number of CTBP2+PSD95+ puncta in the two groups. Experiments were performed in independent experiments (Data are shown as mean ± SEM. n = 3. * Indicates *p* < 0.05. ** indicates *p* < 0.01, *** indicates *p* < 0.001).

Stem cell based therapy had emerged as a promising approach to the treatment of hearing loss. Native functional hair cells are innervated by SGNs, while the regenerated organoid hair cells lack SGNs innervation. Although we improved hair cell differentiation efficiency by incorporating MXene into Matrigel, it was still unknown whether these regenerated organoid hair cells could establish innervation to SGNs of modiolus. In this work, co‐culture methods had been designed to investigate their synaptic reconstruction abilities in a 3D environment in vitro (**Figure** **7**a). Cochlea cells were seeded in 3D substrates for 10 days in a FCM. Subsequently, the medium was changed to a differentiation medium for another 5–7 days of culture. Organoids were then separated from hydrogel and seeded with modiolus on hydrogel precoated wells. After hydrogel solidification, Cochlea‐Orgs and modiolus were covered by additional hydrogel in 3D environments. BF images (Figure 7a and Figure S3, Supporting Information) and confocal images (Figure 7b,c) showed that the Cochlea‐Orgs were surrounded by SGNs after 7 days of co‐culture. SGNs extended out in the 3D hydrogel and surrounded Cochlea‐Orgs, making it promising to establish synapses with organoid hair cells. Immunofluorescence results showed that many regenerated Myo7a+ hair cells in the MXene‐Matrigel group and the Matrigel group (Figure 7b,c) were surrounded by Tuj1+ SGNs, suggesting that synaptic connections were successfully established between regenerated hair cells and SGNs.

To verify synaptic connections were established between hair cells and SGNs, PSD95 and CTBP2 co‐staining experiments were conducted. PSD95 is one of the most commonly used post‐synaptic markers of SGNs, and CTBP2 is the most commonly used pre‐synaptic marker of hair cells. The formation of synapse‐like contacts between cochlea hair cells and SGNs was observed by immunofluorescence and further proved by the co‐staining of CTBP2‐PSD95 puncta (Figure 7d,e). Since Myo7a antibodies were not available for the co‐staining synapse, Atoh1‐GFP mouse line was used to trace regenerated hair cells. The statistical results demonstrated that the indicated CTBP2+ PSD95+ hair cells in the MXene‐Matrigel group were significantly higher than those in the Matrigel group (Figure 7f). Furthermore, it also demonstrated that the number of CTBP2+PSD95+ puncta of the MXene‐Matrigel group was significantly higher than that of the Matrigel group (Figure 7g). These data illustrated that regenerated organoid hair cells can establish innervation with SGNs and the MXene‐Matrigel composites promoted the formation efficiency of CTBP2+ PSD95+ synapse‐like contacts between hair cells and SGNs.

## 3 Discussion

The extracellular matrix (ECM) in various tissues takes the shape of various nanostructures to enable tissue growth, such as bone tissues. Nanomaterials with a size range of hundreds of nanometers can be applied to 3D scaffolds to imitate the ECM of natural tissues to potentiate stem cell proliferation or differentiation by providing complex signaling.^[^ ^42^ ^]^ 3D scaffolds containing nanofibers, nanotubes, and nanoparticles comprised of polymers such as PLA, PLGA, polyvinyl alcohol (PVA), and polycaprolactone (PCL) were discovered to be helpful in affecting stem cell destiny.^[^ ^43^ ^]^ Hydrogels incorporated with nanoparticles to modulate electrical conductivity and mechanical strength, such as carbon nanotube, reduced graphene, gold nanowire, and other conductive nanofibers have been reported.^[^ ^44^ ^]^ Herein, Ti_3_C_2_T_x_MXene, a new material with abundant surface functional groups, is hybrid with Matrigel to form a 3D culture matrix. In this study, 3D MXene‐Matrigel exhibited good biological compatibility up to 300 µg mL^−1^. Others stated that MXene concentrations are safe up to 180 µg mL^−1^ for human periodontal ligament cells and safety up to 500 µg mL^−1^ for HaCat cell line in 2D culture system.^[^ ^26^, ^45^ ^]^ Furthermore, Ti_3_C_2_Tx is a potential material for tissue restoration following tumor surgery due to its great selectivity against tumor cells compared to normal cells.^[^ ^46^ ^]^ In addition, MXenes have an outstanding electrical conductivity of ≈15 100 S cm−1 for a 214 nm thick film,^[^ ^22^ ^]^ making it promising to adjust the conductivity of Matrigel. Matrigel exhibits liner behavior in different manners,^[^ ^47^ ^]^ whereas incorporation of MXene displays nonlinear stress‐strain curves. Another illustrated nonlinear behavior of PI/Ti_3_C_2_Tx and MXene/CNT hybrid aerogels.^[^ ^48^ ^]^ It illustrated that incorporation of materials to Matrigel would adjust the linear behavior of Matrigel. In our previous work, MXene potentiates the hydrophilicity of laminin.^[^ ^49^ ^]^ This isn't surprising that MXene also increases the hydrophilicity of Matrigel.

Taking advantage of biocompatibility and mechanical robustness, carbon‐based materials, including graphene, are an ideal choice for tissue engineering. Carbon‐based scaffolds have been shown to increase cell–cell interaction and normal cellular activities in the clonal culturing of stem cells by exhibiting unique features that are compatible with the natural ECM.^[^ ^50^ ^]^ For constructing tissue structures of different organs, 3D‐graphene scaffolds performed significantly better than 2D scaffolds tested on diverse types of cells, including stem cells derived from the heart, liver, and nervous system.^[^ ^51^ ^]^ In our previous research, we found that Ti_3_C_2_T_x_MXene film does not affect the proliferation of neural stem cells, but enhances the differentiation of neural stem cells with longer neurites and a larger number of branch points and branch tips in neurons.^[^ ^24^ ^]^ In this study, we find that the composite hydrogel containing a certain concentration of Ti_3_C_2_T_x_MXene was suitable for the maintaining and long‐term culture of Cochlea‐Orgs. Our results show that MXene‐Matrigel does not apparently affect the formation rate of Cochlea‐orgs, nor affect cell proliferation of Cochlea‐Orgs. The results are further verified by the CCK‐8 assay and qPCR experiment, confirming that the MXene‐Matrigel matrix does not change the proliferation ability of Cochlea‐Orgs. Notably, we prove that the proportion of Myo7a+ cells in the Cochlea‐Orgs cultured in the MXene‐Matrigel hydrogel is higher than control by immunofluorescence. The expression level of some hair cells specific genes in the MXene‐Matrigel group is significantly higher than in the Matrigel group. Furthermore, transcriptome differences between organoids cultured in Matrigel and MXene‐Matrigel are also investigated by mRNA‐seq. Through GO enrichment analysis, some important signal pathways or cell components have been discovered for the differentiation of Cochlea‐Orgs. These data confirm that MXene‐Matrigel promotes the cochlea stem cells to differentiate into hair cells. Another demonstrates that Ti_3_C_2_T_x_MXene promotes differentiation human mesenchymal stem cells.^[^ ^52^ ^]^

Graphene oxide could enhance the differentiation abilities of several lines of stem cells.^[^ ^53^ ^]^ Chen et al. claim that graphene oxide promotes osteogenic differentiation via MAPK signaling pathway. Whereas Cui et al. have demonstrated that Ti_3_C_2_T_x_MXene induces distinguish differentiation of human periodontal ligament cells by activating WNT/HIF1‐*α* signaling pathway.^[^ ^26^ ^]^ Through GO enrichment analysis on mRNA‐seq data, we find cell response to interferon‐*β* in top 10 GO terms has been significantly up‐regulated. Interferon‐*β* activity has been reported to be regulated by mTOR signaling.^[^ ^54^ ^]^ Hundreds of IFN‐stimulated genes are synthesized when Type I interferon (Interferon‐*α*/*β*) binds to the cell surface receptors IFNAR1 and IFNAR2.^[^ ^55^ ^]^ Moreover, it has been demonstrated that mTOR activity is essential to the formation^[^ ^14^ ^]^ and long‐term survival of hair cells.^[^ ^31^ ^]^ Taken together, we hypothesize MXene‐Matrigel modulates the generation of organoid hair cells by activating mTOR signaling. Immunoblots show that protein levels of P‐AKT, P‐GSK3*β*, and P‐S6 are accumulated in the presence of MXene, confirming that mTOR signaling pathway is potentiated. It is further verified by P‐S6 immunofluorescent labeling. And mTOR activity regulated by agonist or antagonist apparently affects hair cell differentiation in our work. Li et al. also illustrate that de‐differentiation of auditory supporting cells was controlled by mTOR activity.^[^ ^14^ ^]^

Cochlea‐Orgs derived from mouse cochlea stem cells successfully generated cochlea‐like hair cells, which is verified by immunofluorescent and morphology analysis.^[^ ^12^ ^]^ In another work, Cochlea‐Orgs derived from human iPSC can only generate functional utricle‐like hair cells, but failed to induce and differentiate into cochlea‐like auditory hair cells.^[^ ^13^ ^]^ Whereas, whether the regenerated cochlea hair cells in organoids are functional has not been well investigated. Here, the function and maturity of newly generated organoid hair cells were tested by recording their electrophysiological properties. These newly generated hair cells in the MXene‐Matrigel group were closer to native hair cells than in the Matrigel group according to electrophysiological properties. The RMP for organoid hair cells cultured in the MXene‐Matrigel substrates was significantly lower than the Matrigel group. Second, the statistical results showed that the calcium currents of the MXene‐Matrigel group were significantly larger than the Matrigel group (31.57±13.01pA). The function and maturity of newly generated organoid hair cells were tested by recording their electrophysiological properties. The potassium current in the MXene‐Matrigel group (about 2nA) was smaller than P4 utricle (about 6nA).^[^ ^40^ ^]^ It seems like the maturity of MXene‐Matrigel group might be prior to native utricle hair cells at P4. Thus, we've already conducted the experiment about the current and voltage response for cochlear hair cells and vestibular hair cells at P2 showing the current response of the Matrigel group was similar to the native IHCs at P2, with slightly lower than the MXene‐Matrigel group. And the voltage response (membrane potential) of the MXene‐Matrigel group was quite similar to those of the native cochlear hair cells and vestibular hair cells at P2. Liu. et al. had the same results for P2 vestibular hair cells. Collectively, the maturity of regenerated organoid hair cells in our MXene‐Matrigel group is likely to be perinatal at P2‐4 of native IHCs.

Organoids, while made up of several cell types and cultivated in a 3D environment, lack essential microenvironmental signals such as sympathetic and parasympathetic innervation as well as immune cells. These are important variables in tissue formation and regeneration. The significance of parasympathetic innervation in tissue development^[^ ^56^ ^]^ and regeneration,^[^ ^57^ ^]^ is becoming more clear. Finding a way to properly imitate the neurogenic innervation of organoids is necessary before they can be adequately used as models for the regeneration of tissues with similar architecture to native organs or tissues. Previously, we have discovered that MXene potentiates differentiation of neural stem cells,^[^ ^49^ ^]^ making it promising to promote neuron innervation by incorporating of MXene. In this work, we successfully establish synaptic connections between the newly generated organoid hair cells and the SGNs of the Cochlea modiolus in vitro. By co‐staining the presynaptic CTBP2 of hair cells and postsynaptic PSD95 of SGNs, we calculate the co‐labeling of CTBP2 and PSD95, and successfully record the establishment of new synaptic connections between regenerated hair cells and SGNs. However, the formation of CTBP2 + and PSD95 + puncta is still at low efficiency than that in the cochlea sensory epithelium.^[^ ^13^ ^]^ This suggests that although synaptic connections between hair cells and SGNs can establish in vitro, the synapse connections between newly generated hair cells and SGNs are still not enough. Furthermore, we realize that there would be a considerable difference in the regenerate organoid hair cells, especially for innervated hair cells. Unfortunately, we fail to record electrophysiological features of organoid hair cells innervated by SGNs in our co‐culture system. It is still obscure whether neuronal innervation could be beneficial for hair cell maturation or function. How to further promote the formation of synaptic connections between hair cells and SGNs will be key in the future.

In this study, we demonstrated that MXene‐Matrigel potentiates differentiation and maturation of Organoid hair cells. To our knowledge, there is little study illustrating that MXene hybrid gels are found to promote the maturation and differentiation of organoids. In previous studies, we have shown that 2D Ti3C2TxMXene films stimulate neural stem cell differentiation.^[^ ^49^ ^]^ A recent study found that WNT/HIF1‐*α* plays a role in the regeneration and differentiation of human periodontal ligament cells.^[^ ^26^ ^]^ Here, we demonstrated that incorporation of Ti_3_C_2_T_x_MXene to Matrigel can promote hair cell differentiation via mTOR signaling pathway. Furthermore, MXene‐Matrigel also benefits the formation of innervation between regenerated hair cells and SGNs.

## 4 Conclusion

In summary, we prepared MXene composite hydrogel by incorporating Ti_3_C_2_T_x_MXene into Matrigel and investigated its effects on the pluripotency of Cochlea‐Orgs. First, we verified the properties of Ti_3_C_2_T_x_MXene through characterization and further examined whether Ti_3_C_2_T_x_MXene could improve the physicochemical properties of Matrigel. Second, we found that the MXene‐Matrigel hydrogel maintained the proliferation of Cochlea‐Orgs and significantly promoted their differentiation abilities. Then, organoid hair cells derived from cochlea stem cells in the MXene‐Matrigel group displayed better electrophysiological features comparable to the Matrigel group and comparable to native P2 cochlea inner hair cells. Furthermore, we found that MXene‐Matrigel can potentiate hair cell differentiation by activating mTOR signaling. Finally, we successfully developed an initial co‐culture system for Cochlear‐Orgs and modiolus to establish innervation of hair cells. And by using the MXene‐Matrigel composite hydrogel, the formation efficiency of synaptic connections between organoid hair cells and SGNs was significantly increased.

## 5 Experimental Section

### Mice

The Southeast University Institutional Animal Care and Use Committee procedure was followed for all animal research. The wild‐type C57BL/6 mouse line and Lgr5‐EGFP‐IRES‐Cre‐ER mice (The Jackson Laboratory, strain 8875) were used for initial organoid culture and modiolus co‐culture. The Atoh1‐EGFP mouse line, also known as Math1^M1GFP/M1GFP^ mouse line (The Jackson Laboratory, strain 01 3593) was used to trace newly generated hair cells. To efficiently isolate cochlear stem cells from the cochlear basement membranes, mice around p2 were used as described before.^[^ ^58^ ^]^

### Synthesis of Ti_3_C_2_TxMXene

The protocol for Ti_3_C_2_TxMXene synthesis has been described before.^[^ ^24^ ^]^ Briefly, multilayer Ti_3_C_2_ was produced by etching Ti_3_AlC_2_ in a mixture of HCl and LiF. After the reaction was completed, the solution was centrifuged and rinsed until it reached a pH of 6.0 using deionized water. The Ti_3_C_2_ was then sonicated for 1 h in an ice bath before being combined with deoxidized and deionized water. The Ti_3_C_2_TxMXene was stored at 4 °C.

### Characterization of Ti_3_C_2_TxMXene and MXene‐Matrigel

MXene was diluted to 250 µg mL^−1^ by water. Droplets from the solution were left to dry on a carbon‐coated copper grid (200 mesh).^[^ ^59^ ^]^ And Images of TEM were obtained by using a Talos F200X electron microscope. And SEM images were acquired by a SU8010 electron microscope. The Ti_3_C_2_TxMXene film was prepared with an Air Cantilever in RT for the following tests. A Smartlab 9kw X‐ray diffractometer was used to record the XRD pattern. The XPS spectra were recorded using a Thermo Fisher Nexsa X‐ray photoelectron spectrometer, while the Raman spectra were measured using a Thermo Fisher is50 Fourier transform infrared spectrometer. The water contact angles were recorded on a JY‐82B Kruss DSA contact angle meter in RT. The conductivity of MXene‐Matrigel and Matrigel were shown with an ST2422 resistivity tester.

### Isolation of Cochlea Cells from Mouse Inner Ear

HBSS (Wisent Corporation, no. 311‐512‐CL) was pre‐cooled in a refrigerator at 4 °C. The scissors and tweezers were immersed in 75% alcohol and exposed to UV for 30 min. The auditory epitheliums were carefully dissected from around P2 mouse cochlea and placed in clean pre‐cooled HBSS. After washing at 500 rpm for 5 min, Type IV collagenase (Sigma‐Aldrich, no. C4‐28) and Ca2+ were added and incubated at 37 °C for 5 min for digestion. After digestion, 1 mL pre‐cooled advanced DMEM/F12 (AdDMEM/F12) medium was added (Gibco, no. 12 634 010) and centrifuged at 1000 rpm for 3 min. In the following step, dissociated cells were filtered through a 40 µm filter (Falcon, no. 352 340) and centrifuged for 3 min at 1000 rpm. After discarding the medium, an appropriate amount of fresh medium was added to resuspend the cells.

### Organoid Culture of Cochlear Stem Cells

Cells isolated from theauditory epitheliums were washed twice with pre‐cooled advanced DMEM/F12. After counting, cells were mixed with growth factor reduced Matrigel (R&D systems, no. 3533‐005) and MXene‐Matrigel in collection tubes. After the substrates were solidified at 37 °C in cell incubator (ThermoFisher, no. BB150‐2TCS), FCM was added. FCM was composed of AdDMEM/F12, with GlutaMax (Gibco, no. 35 050 061), Primocin (Invivogen, no. ant‐pm‐1), 10 µm HEPES (Gibco, no. 15 630 080), 1:100 B27 (Gibco, no. 12 587 010), 1:50 N2 (Gibco, no. 17 502 048), 50 ng mL^−1^ EGF (Peprotech, no. 100–15), 50 ng mL^−1^ FGF (Peprotech, no. 100–18b); 3 µm CHIR99021 (Tocris, no. 4423); 1 mm VPA (Sigma‐Aldrich, no. P4543), 100 µg mL^−1^ pVc (Sigma‐Aldrich, no. 49 752), and 1 mm A83‐01 (Tocris, no 2939).

Cochlea‐Orgs were cultured as previously described.^[^ ^8^ ^]^ During organoid expansion, FCM was changed every 2 or 3 days. FCM was replaced by differentiation medium 10 days after proliferation. The differentiation medium was comprised of AdDMEM/F12, primocin, 1:100 B27, 1:50 N2, 3 µm CHIR99021, and 5 µm LY411575 (MedChemExpress, no. HY‐50752). The media was replaced every 2 or 3 days during the differentiation of Cochlea‐Orgs.

### EdU Staining

EdU staining was conducted according to the manufacturer's instructions using the Click‐iT EdU imaging kit (Invitrogen, no. C10337). For organoid proliferation studies, EdU was diluted by FCM and incubated with organoids for 3 h. After fixation, Cochlea‐Orgs were washed with PBST (0.1% TritonX‐100) for 3 times. 60 µL reaction solution was added (for each well) and incubated at RT away from light for 30 min. The reaction solution was sucked up and washed 3 times with PBST. Samples were incubated with DAPI (Solarbio Life Sciences, no. C0065) at RT for 1 h to label the nucleus. Confocal images were obtained on an LSM700 or LSM900 confocal microscopy (Zeiss).

### CCK‐8 Assay

CCK‐8 assay was performed following the manufacturer's instructions. After 7 days of expansion, CCK‐8 solution (Beyotime, no. C0038) was added to fresh FCM and the cells were incubated for 3 h in the cell incubator. The absorbance was measured with a Cytation 5 Multi‐Mode reader (BioTek) at 450 nm after an equivalent volume of medium was transferred into a 96‐well plate.

### RNA Isolation and qRT‐PCR

Following manufacturers' instructions, RNA was isolated from Cochlea‐Orgs using RNeasy Mini Kit (Qiagen, no. 74 104), and reverse transcription was performed by using RevertAid First Strand cDNA Synthesis Kit (ThermoFisher, no. K1622). qPCR analysis was carried out in a 96‐well qPCR machine (Bio‐Rad Laboratories, CFX96) using SYBR Green Mixture (Vazyme, no. Q712‐02). Primers for qPCR were described before^[^ ^8^, ^14^ ^]^ or checked through Primer‐BLAST and are listed in Table S1, Supporting Information.

### mRNA Sequencing and Analysis

Total mRNA was extracted using the QIAGEN RNeasy Mini kit or TRIzol (Invitrogen, no. 15 596 018) and reverse transcribed with the reverse transcription kit. Transcription was acquired by using the appropriate amount of cDNA template and reversed into the sequencing library. Samples were sequenced using Illumina NovaSeq 6000. PCA was used to evaluate sample variability. R packages (4.2.0 version) were used to implant RNA‐seq data. As for PCA algorithm, these SEQ data were interplayed for DEseq2 downstream analysis. In detail, count values were initially normalized by “vst” algorithm that was widely used for removal of the dependence of the variance on the mean, particularly the high variance of the algorithm of count data when the mean was low, as well as the removal of the experiment‐wide trend. Then “plotPCA” function was used to visualize the PCA distribution. Differential genes were analyzed using the DeSeq2 package for visual heat map analysis.

### Immunohistochemistry, Immunofluorescence, Whole Mount Staining, and Microscopy

Cell recovery solution (Corning, no. 354 253) was used to harvest organoids, which were then fixed in 4% PFA at RT for 30 min. The organoids were then washed with PBST, and permeabilized in PBST, followed by 1 h of blocking at RT with 10% donkey or goat serum. Primary antibodies were incubated overnight at 4 °C. The next day, organoids were washed with PBST and incubated with secondary antibodies, Phalloidin (Invitrogen, no. A12379 or A34055), and DAPI for 1 h at RT. A Zeiss LSM 700 or LSM900 confocal microscope was used for organoid imaging. Images processing was conducted by Adobe illustrator, ImageJ software, and Zen imaging software. Primary antibodies and second antibodies are listed in Table S2, Supporting Information.

### Co‐Culture of Organoids and Modiolus

Cell recovery solution was used to harvest organoids, which were then placed in ice‐cooled HBSS. Modiolus were dissected from mouse cochlea and washed twice with precooled AdDMEM/F12. Organoids were mixed with Matrigel and MXene‐Matrigel and seeded into 24 well‐plates. Then, an equal amount of modiolus was seeded around Cochlear‐Orgs under stereoscopes. Organoids and modiolus were cultured in the differentiation medium (described above) and the medium was changed every 2 or 3 days.

### Electrophysiological Recordings

The conventional whole‐cell patch‐clamp recordings were made from P2 native inner hair cells, P2 mouse utricle hair cells, and Atoh1‐GFP inner ear organoids cultured in the Matrigel or Mxene‐Matrigel substrates on d30. Cells were bathed and recorded in artificial cerebrospinal fluid containing 126 mm NaCl, 4.9 mm KCl, 1.2 mm KH2PO4, 2.4 mm MgSO4, 2.5 mm CaCl2, 26 mm NaHCO3, and 10 mm glucose at pH 7.4 bubbled with 95% O_2_/5% CO_2_ at room temperature. Cells were visualized under upright microscopy (Olympus) equipped with water immersion lenses (60×) to view and target cells, then recorded with Axopatch‐1550B amplifier (Molecular Devices) and analyzed using pCLAMP10 software (Molecular Devices). Recording pipettes were pulled from borosilicate glass capillaries (0.86/1.5 mm: ID/OD) and filled with an intracellular solution consisting of 136 mm K‐gluconate, 6 mm KCl, 1 mm EGTA, 2.5 mm Na_2_ATP, and 10 mm HEPES (280 mm mOsm, adjusted to pH = 7.2 with KOH). Calcium current (*I*_ca_) were recorded by a Cs‐based internal solution containing 135 mm Cs‐methane sulfonate (Sigma‐Aldrich, C1426), 10 mm CsCl, 10 mm TEA‐Cl (Abcam, ab120275), 2 mm EGTA, 10 mm HEPES, 3 mm Mg‐ATP, and 0.5 mm Na‐GTP (290 mm mOsm, adjusted to pH = 7.2 with CsOH). Cochlea inner hair cells, utricle hair cells, and organoid hair cells were held at −50 mV. Firing was elicited with a series of current steps (10 steps of 10 pA increments) duration in current‐clamp mode. Recordings were performed in voltage‐clamp mode, applying from −100 mV with an increment of 10 mV in voltage steps of 500 ms duration. Calcium currents (*I*_ca_) were recorded in response to a voltage ramp under voltage clamp from −87 to + 63 mV in 150 ms. For all recordings, the leak currents were subtracted using the P/4 procedure. Cells displaying a leak current >100 pA were discarded. Data were low‐pass filtered at 2 kHz and acquired at 5–10 kHz. Recordings were low‐pass filtered at 2 kHz. Data analyses were performed using Clampfit and GraphPad software.

### Explant Culture

P2 mouse cochlear was isolated and collected in HBSS. The auditory epithelia were dissected from cochlea and attached to slides by Cell‐Tak (Corning, no. 354 240). The explants were cultured in AdDMEM/F12, penicillin (100 U mL^−1^), N2 (1:100), B27 (1:50), CHIR99021 (3 µm), and LY411575 (5 µm). To potentiate mTOR signaling, wild‐type or Atoh1‐EGFP + cochlear explants were treated with 10 µm HYM1485 (MedChemExpress, no. HY‐B0795) or vehicle control DMSO. To inhibit mTOR signaling, wild‐type or Atoh1‐EGFP + cochlear explants were treated with 10 µm Rapamycin (MedChemExpress, no. HY‐10219) or vehicle control DMSO. The culture media were exchanged every other day, and cultures were maintained for 4 days.

### Western Blotting

Cochlea‐Orgs and cochlear auditory epithelia were lysed in a collection tube by total Protein Extraction Kit (Sangon Biotech, no. C510003‐0050). The proprotein concentration was measured using a BCA kit (Beyotime, no. P0010). Samples were loaded into 10% PAGE gels (Vanzyme, no. E303‐01). After being incubated with 5% BSA at RT, samples were probed by anti‐ HIF1‐*α* (Proteintech, no. 20960‐1‐AP), Anti‐*β*‐catenin (BD, no. 610 153), anti‐P‐S6 (Ser235/236) (CST, no. 4857S), anti‐S6 (CST, no. 2217S), ant‐Akt (CST, no. 9272S), anti‐P‐Akt (Ser473) (CST, no. 4060P), anti‐ GSK3*β* (CST, no. 12456S), anti‐ P‐GSK3*β* (CST, no. 5558T), and an anti‐rabbit/mouse HRP‐conjugated secondary antibody.

### Statistical Analysis

Results were presented as mean ± SD or mean ± SEM from independent experiments carried out in the same manner. The statistical analyses were performed by GraphPad Prism 9 with two‐tailed, unpaired Student's t‐test (n ≥ 3). Photoshop and Image J were used to calculate protein levels and cell numbers. GraphPad Prism 9 or Origin Pro was used for graphing. For all tests, *, **, and *** stand for *p* < 0.05, *p* < 0.01, and *p* < 0.001, respectively.

## Conflict of Interest

The authors declare no conflict of interest.

## Supporting information

Supporting Information

## Data Availability

The data that support the findings of this study are available from the corresponding author upon reasonable request.
