# Supplementary material for: Ti3C2TxMXene Composite 3D Hydrogel Potentiates mTOR Signaling to Promote the Generation of Functional Hair Cells in Cochlea Organoids
Source: Adv Sci (Weinh). 2022 Sep 18;9(32):2203557. doi: 10.1002/advs.202203557 (PMC9661825; doi:10.1002/advs.202203557)
Supplement: Supplementary file 1 — Supporting Information [file ADVS-9-2203557-s001.pdf]

ADVANCED SCIENCE
Open Access

Supporting Information

for *Adv. Sci.*, DOI 10.1002/advs.202203557

$Ti_3C_2T_x$MXene Composite 3D Hydrogel Potentiates mTOR Signaling to Promote the Generation of Functional Hair Cells in Cochlea Organoids

*Zhong Zhang, Shan Gao, Yang-Nan Hu, Xin Chen, Cheng Cheng, Xiao-Long Fu, Sha-Sha Zhang, Xin-Lin Wang, Yu-Wei Che, Chen Zhang and Ren-Jie Chai**

Supplementary Materials

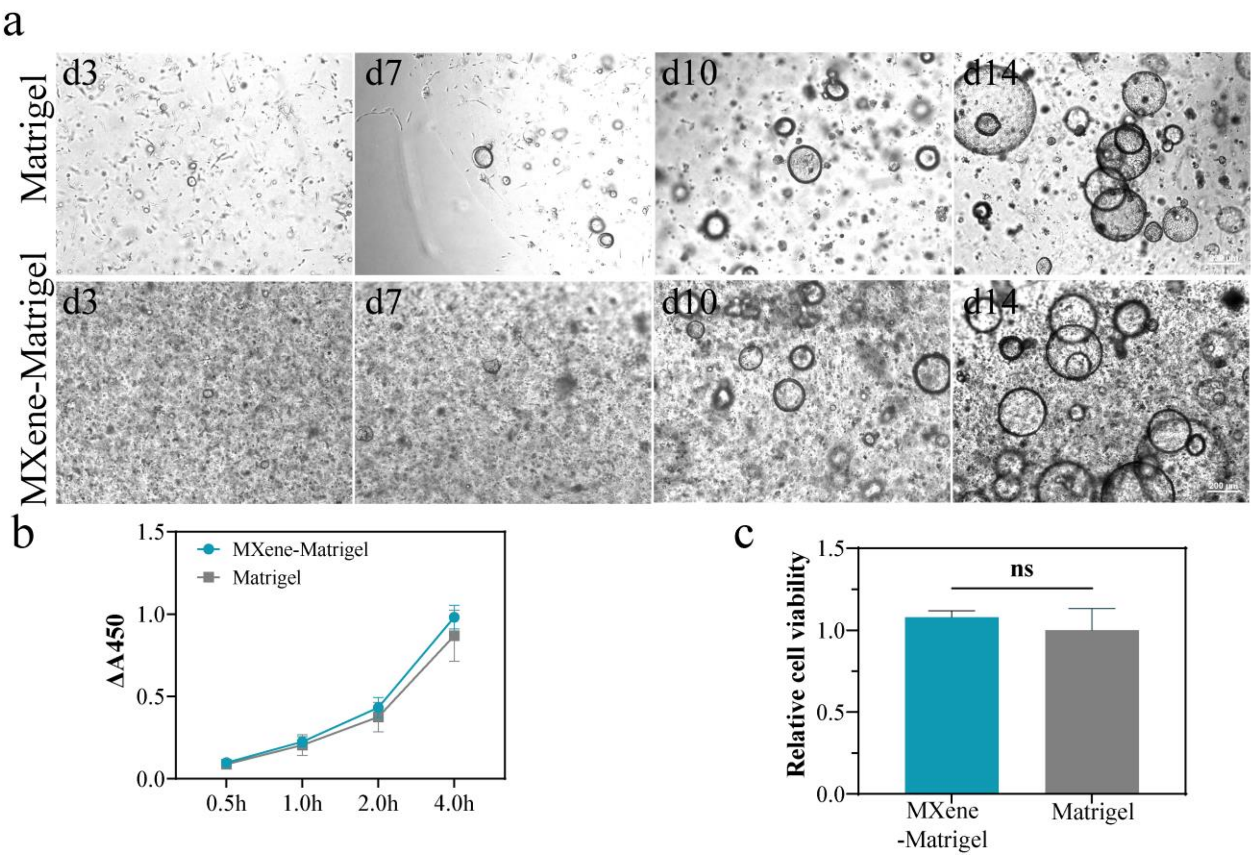

Figure s1. Development of Cochlea-Orgs in different time points. a) Representative BF images of Cochlea-Orgs development in MXene-Matrigel and Matrigel. Small pellets were formed at day3 and develop into organoids at day 7. After further expansion, the diameter of Cochlea-Orgs became bigger at day10 and day14. b, c) After 10 days of expansion, the relative cell viability of the Mxene-Matrigel and Matrigel groups was tested by conducting CCK-8 assay. A450 was measured to determine the relative cell viablity in the two groups at different time points.

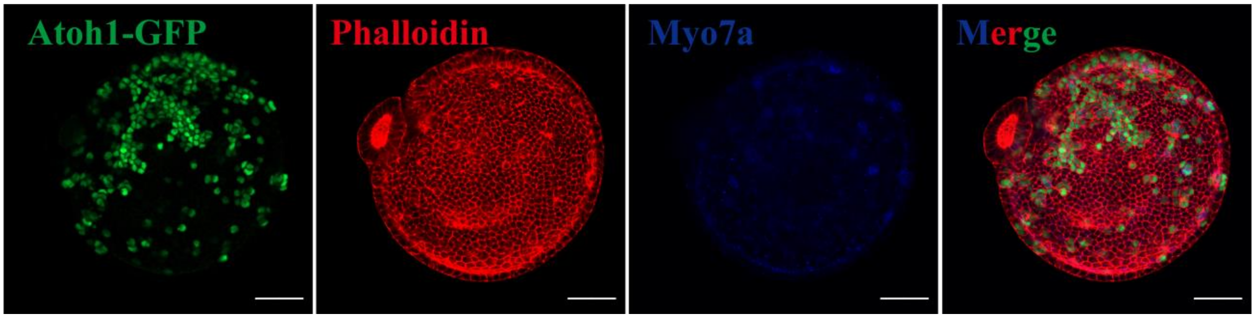

Figure s2. The expression of Atoh1-GFP and Myo7a in organoid cultured in FCM. After proliferation for 10 days in Mxene-Matrigel, most of newly generated hair cells are labeled by Atoh1-GFP+ hair cells but not Myo7a. Scale bar = 50 μm.

a

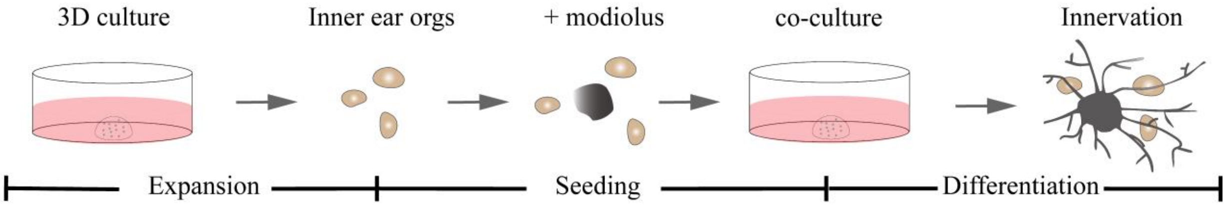

b

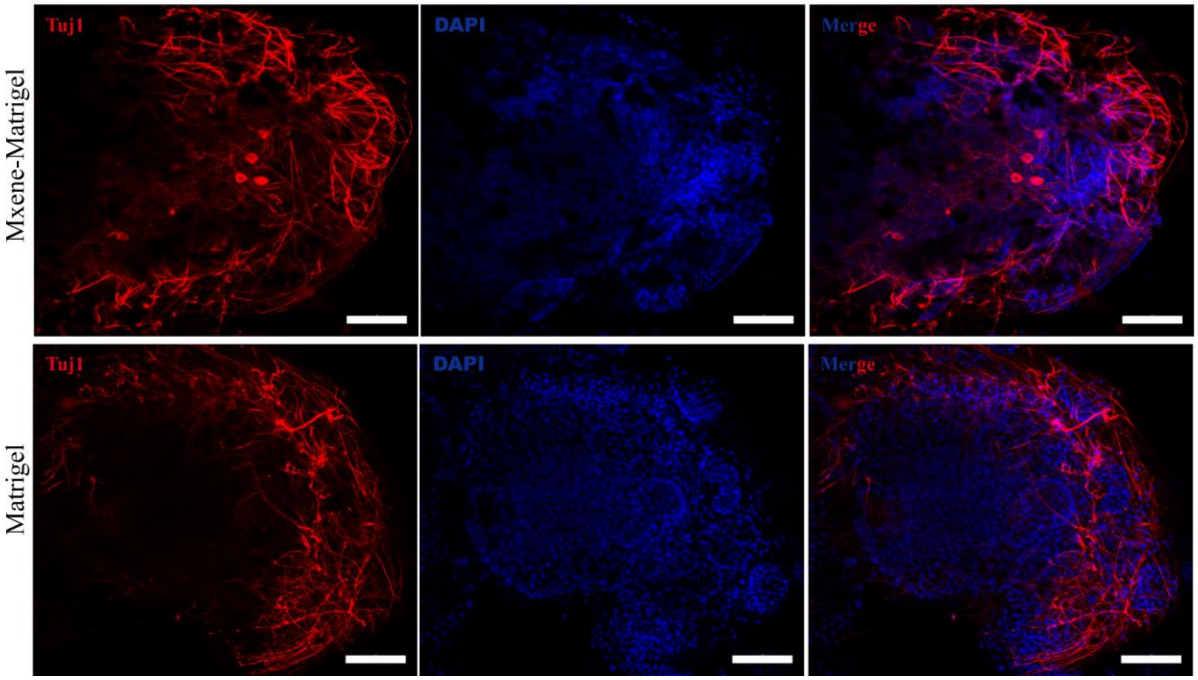

Figure S3. Cochlea-Orgs and modiolus co-culture. a) Experimental design for 3D co-culture system of Cochlea-Orgs and modiolus. b) immunofluorescent images showed the connection of Cochlea-Orgs and modiolus in the co-culture system. Scale bar = 100 μm.

Table S1. The list of primer sequences

Atoh1	Forward	ATGCACGGGCTGAACCA
	Reverse	TCGTTGTTGAAGGACGGGATA
Myo7a	Forward	CCCCCTCTGAGAAGTTCGTTAA

	Reverse	TGTGTCCGAGTTCCGTTGAC
vGlut3	Forward	TGGACCTTCTATTTGCTCCTG
	Reverse	GCACCACGATTGTCATCACC
Pou4f3	Forward	GCACCATCTGCAGGTTCGA
	Reverse	CCGGCTTGAGAGCGATCAT
Ano1	Forward	TTCCCTCTGGCTCCACTCTTC
	Reverse	GGCATCCAGGCGGATCT
Kncn	Forward	GGCTCCTCATCTTCGCCTAC
	Reverse	CCATGGTCTGGTCCTGAGTT
cav1.3	Forward	TGCACAGATGAAGCCAAAAG
	Reverse	TGTCCCATCACTGTTGAGAGGCAT
Kcnq	Forward	CTTGGCTGGGAAGATTGCTTTC
	Reverse	TCTCAAAGTGTTTCTGGCGGTG
Kcnc10	Forward	GAGACCAGCACATCCCATCT
	Reverse	TAGCCTGGCTCTTCATGGAT
Myo3b	Forward	TGCCTCCACATTTCAGTGCTGG
	Reverse	CGACCACCATTTTGGAGAGCAG
Cdh23	Forward	ACACCAGTGGGGACACCCATCTTCATC
	Reverse	AGACAATAGTGTAGCCAATCCCACGG
Ptprq	Forward	GTATGGCTGACGTGGAGTCCAA
	Reverse	GTCCAGCAAGTTTGGCATCAGTC
Loxhd	Forward	TACGAGGAGGAGCTGCTGAACTACGA
	Reverse	CATCCCCTGTGGCTGTGACC
Wif1	Forward	CTTTGCTGGGAACAGTGCCTCA

	Reverse	GGTCCTAAGGATGGTGTTGCCT
Dkk3	Forward	GGAGGAAGCTACGCTCAATGAG
	Reverse	TTGCCAGGTTCACCTCAGAGGA
Oct	Forward	GCCTTGCAGCTCAGCCTTAA
	Reverse	AAGCCAGGAATGGAAGCAGC
Sox2	Forward	CCAGCGCATGGACAGCTA
	Reverse	GCTGCTCCTGCATCATGCT
Pax2	Forward	GACAGCACCAGACAAGAGGC
	Reverse	TAGCCAAAAGCCTCGGCAG
Pax8	Forward	CTTTGCAGTCCCCAGCTCAG
	Reverse	GCCAAGTGCTCTCCTGTGTC
Lgr5	Forward	CCCCAATGCGTTTTCTACGT
	Reverse	GAAGGACGACAGGAATTGGAT
Fst	Forward	GAAAACCTACCGCAACGAATG
	Reverse	TCCGGCTGCTCTTTGCAT
Hmga2	Forward	CAGAAGAAAGCAGAGACCATT
	Reverse	TTGTTGTGGCCATTTCCTAGGT
Fat3	Forward	CACAGCCCTTGAATACAGTGA
	Reverse	TGCCTTTGCATCTCCTTCCT
Trim71	Forward	ATCGGGAGTGTGAGCTGTTG
	Reverse	GGCGTGAACATAATGCGGTC
Nestin	Forward	GTCCTGGTTCCTGAACTTGTC
	Reverse	GCTTCTTTCTCTACCAGTTCCC
GAPDH	Forward	AACACAGTCCATGCC

	Reverse	TCCACCACCCTGTTGCTG

Table S2. List of antibodies used for immunostaining.

Reagent type	Designation	Source	Identifiers
primary antibody	myo7a Rabbit polyclonal	Proteus Biosciences	Cat.# 25-6790
primary antibody	SOX2 Goat polyclonal	Santa Cruz	Cat.# sc-17320
primary antibody	βIII Tubulin Mouse monoclonal	R&D systems	Cat.# MAB1195
primary antibody	PSD95 Mouse monoclonal	Sigma-Aldrich	Cat.# MAB1596
primary antibody	Synapsin-1 (D12G5) Rabbit monoclonal	Cell Signaling	Cat.# #5297
secondary antibody	donkey anti-rabbit IgG (H+L) Alexa Fluor 555	Thermo Fisher	Cat.# A-31572
secondary antibody	donkey anti-mouse IgG (H+L) Alexa Fluor 488	Thermo Fisher	Cat.# A-21202
secondary antibody	donkey anti-goat IgG (H+L) Alexa Fluor 647	Thermo Fisher	Cat.# A-21447
secondary antibody	goat anti-mouse IgG2a Alexa Fluor 647	Thermo Fisher	Cat.# A-21241
secondary antibody	goat anti-rabbit IgG (H+L) Alexa Fluor 546	Thermo Fisher	Cat.# A-11011
